# Sex Differences in the Association between Night Shift Work and the Risk of Cancers: A Meta-Analysis of 57 Articles

**DOI:** 10.1155/2018/7925219

**Published:** 2018-11-26

**Authors:** Wen Liu, Zhonghan Zhou, Dahai Dong, Lijiang Sun, Guiming Zhang

**Affiliations:** Department of Urology, The Affiliated Hospital of Qingdao University, Qingdao, China

## Abstract

**Objectives:**

To identify the association between night shift work and the risk of various cancers with a comprehensive perspective and to explore sex differences in this association.

**Methods:**

We searched PubMed, Embase, and Web of Science for studies on the effect of night shift work on cancer, including case-control, cohort, and nested case-control studies. We computed risk estimates with 95% confidence intervals (CIs) in a random or fixed effects model and quantified heterogeneity using the *I* ^2^ statistic. Subgroup, metaregression, and sensitivity analyses were performed to explore potential sources of heterogeneity. Contour-enhanced funnel plots and the trim and fill method were used together to analyze bias. Linear dose–response analysis was used to quantitatively estimate the accumulative effect of night shift work on the risk of cancer.

**Results:**

Fifty-eight studies were eligible for our meta-analysis, including 5,143,838 participants. In the random effects model, the pooled odds ratio (OR) of cancers was 1.15 (95% CI = 1.08–1.22, *P* < 0.001; *I* ^2^ = 76.2%). Night shift work increased the cancer risk in both men (OR = 1.14, 95% CI = 1.05–1.25, *P* = 0.003) and women (OR = 1.12, 95% CI = 1.04–1.20, *P* = 0.002). Subgroup analyses showed that night shift work positively increased the risk of breast (OR = 1.22, 95% CI = 1.08–1.38), prostate (OR = 1.26, 95% CI = 1.05–1.52), and digestive system (OR = 1.15, 95% CI = 1.01–1.32) cancers. For every 5 years of night shift work, the cancer risk increased by 3.2% (OR = 1.032, 95% CI = 1.013–1.051).

**Conclusion:**

This is the first meta-analysis identifying the positive association between night shift work and the risk of cancer and verifying that there is no sex difference in the effect of night shift work on cancer risk. Cancer risk increases with cumulative years of night shift work.

## 1. Introduction

Recent years have witnessed a rise in the number of people working late or night shifts in different employment sectors, such as healthcare, construction, transportation, and food preparation [1, 2]. The rate of shift work can exceed 15% of the workforce in many countries of North America, continental Europe, and Australia [2], and the trend is increasing. Night shift workers not only have higher short-term safety risks because of decreased alertness [3] but also have greater long-term health risks, including for diabetes [4], obesity [5], cardiovascular disease [6], depression [7], and cancer [8]. In 2007, a report by the International Agency for Research on Cancer (IARC) classified night shift work involving circadian disruption as “probably carcinogenic to humans” based on sufficient evidence in animal experiment and limited evidence in humans [9]. Therefore, investigating the influence of night shift work has captured attention. Most previous original studies verified the effect of night shift work on cancer risk, but the results are controversial for different cancers. Some findings have indicated that night shift work is significantly associated with higher cancer risk [10–39] whereas other studies have provided insignificant evidence for this relationship [40–66], motivating further study.

There were several postulated causal mechanisms that explain how night shift work multiplies cancer risk. First, melatonin, a marker of circadian rhythms, has a fundamental impact on inhibiting carcinogenesis through antioxidation, regulation of immunity, free radical scavenging, and antiangiogenesis [67]. Generally, night shift workers have a substantially decreased melatonin level during the nighttime [68, 69]. Melatonin suppression has been reported in breast [69], prostate [70], lung [71], ovarian [67], and gastrointestinal [72] cancers. Second, the 24-hour circadian rhythm is generated via interacting feedback loops of the circadian genes in all cells of both the hypothalamic suprachiasmatic nucleus (SCN) and all peripheral tissues [73, 74]. Night shift work can induce a conflict between the endogenous circadian clock and the external shifted sleep period and feeding behavior, leading to a dampening of the gene expression rhythm (25% of circadian genes) and subsequent disordered expression of transcription and translation in these cells [73, 74]. These disturbances can interfere with cell proliferation, apoptosis, hormonal balancing, metabolism, DNA damage repair, and immune and neuroendocrine functions. Recent studies have uncovered that the disruptive expression of circadian genes especially increases the risk of cancers in the immune, skeletal, digestive, and reproductive systems in which cell proliferation, metabolism, and DNA damage repair are required to maintain daily function [74]. Overall, the mechanisms based on hormonal and molecular levels manifest that the influence of night shift work on cancer is systemic and is not limited to a specific organ. However, many previous meta-analyses have identified the association of night shift work with only one type of cancer, including breast [75–78], prostate [79–81], and colorectal [82] cancers, among others. Only one study [8] analyzed the relationship between night shift work and the risk of cancers in women. Accordingly, we aimed to classify the association between night shift work and the risk of multiple cancers from a comprehensive perspective.

Previous studies have revealed that the circadian timing system differs in the sexes, which is mediated by different neuroendocrine contexts, such as sex hormones and their receptors in SCN [16, 83, 84]. Compared with male sex, female sex has been associated with earlier timing and larger amplitude of melatonin and earlier timing and longer duration of sleep [85, 86]. After night shift work, women showed greater impaired performance in health and cognition compared with men. For example, accuracy, alertness, the amplitude of melatonin, and working memory deteriorate more in women [3, 86, 87], enabling us to understand why female was more susceptible to sleep and wake disturbances after shift work [3]. More intense response to shift work in women reminds us whether the effect of night shift work on cancers varies with different genders.

Consequently, we conducted a meta-analysis to investigate this sex difference. We also expanded upon previous meta-analyses by not only evaluating the association between night shift work and a specific cancer but also estimating whether there was a dose–response relationship between night shift work and the risk of multiple cancers.

## 2. Methods

### 2.1. Search Strategy

We conducted a comprehensive updated search through May 2018 using PubMed, Embase, and Web of Science databases. Two investigators searched for eligible English articles independently. The search terms were “night shift work” or “rotating shift work” or “night work” or “shift work” and “carcinoma” or “neoplasm” or “tumor” or “cancer”. In addition, we manually reviewed the reference lists of articles for additional relevant studies.

### 2.2. Inclusion and Exclusion Criteria

Studies were included if they satisfied the following criteria: (i) the research was a case-control study, cohort study, or nested case-control study; (ii) the exposure of interest was night shift work, and the outcome of interest was the risk of any type of cancer; (iii) the study reported adjusted risk estimates (odds ratio, OR; relative risk, RR; hazard ratio, HR) with 95% confidence intervals (CIs) or provided sufficient data to allow calculation. Studies were excluded if they satisfied the following criteria: (i) the study did not provide sufficient data; (ii) the study mentioned recurrent cancer; (iii) when more than one article was based on the same study population, we only included the study with the largest number of cases.

### 2.3. Data Extraction

Data extraction was conducted independently by two authors for the following items: first author, publication year, study location, study design, number of cases, occupation, quality score, definition of exposure, participant sex, type of cancer, adjusted OR with 95% CI, adjusted covariates, and exposure assessment. As the prevalence of tumor is very low, we considered that ORs equaled RRs or HRs, providing similar risk estimates [88]. According to the definition of work schedule, we divided work schedules into rotating shift (working a regular shift schedule), fixed shift (permanent night work), and mixed (with no clear work schedule). ORs of the longest versus shortest exposure time were extracted from articles as the exposure indicator for statistical analysis. We also extracted dose information from ordinal categorical data (≥3 levels of the exposure category) for dose–response meta-analysis.

### 2.4. Quality Assessment

Two authors performed quality assessment using the Newcastle-Ottawa Quality Assessment Scale (NOS) [89]. The scale comprises a total of 9 points on the three parts of the NOS, including participant selection (0–4 points), comparability (0–2 points), and exposure or outcome assessment (0–3 points). Scores of ≥7 indicate a high quality.

### 2.5. Statistical Analysis

All statistical analyses were performed using Stata version 12.0 (StataCorp, College Station, TX, USA). We preferentially extracted adjusted ORs from original articles to evaluate the association between night shift work and cancer risk. If there were no adjusted ORs for specific subgroup analyses, a number of cases and participants would be extracted to calculate OR. The inverse variance method was used to combine ORs. If *I* ^2^ for the heterogeneity test was ≤50%, a fixed effects model was adopted to pool ORs; otherwise, a random effects model was selected. To explore potential heterogeneity, we performed subgroup analyses, metaregression analyses, and sensitivity analyses. One subgroup analysis was the classification of work schedules; we used a random effects model to evaluate the effect size for cancer on different work schedules [80]. To confirm the stability of results, a sensitivity analysis was conducted by omitting one study and then recalculating the rest of studies. The leave-one-out analysis was used to examine the weight of influence of each study on pooled OR [90].

A generalized least-squares trend (GLST) model was used to estimate the overall dose–response relationship of night shift work and the risk of cancer by computing risk estimates for different ordinal levels of night shift work. There were at least three ordinal levels of the exposure category in each study. The midpoint of the upper and lower boundaries of each level was considered the average exposure. The upper boundary of the highest level was considered the same as the adjacent category if it was not provided [76]. We used a two-stage random effect model to evaluate the linearity between night shift work and the risk of cancer.

Potential publication bias was estimated with the Begg funnel plot. Furthermore, the contour-enhanced funnel plot and the trim and fill method were used together to analyze the cause of bias. All reported *P* values were two-sided, and statistical significance was set at *P* ≤ 0.05.

## 3. Results

### 3.1. Study Selection

**Figure 1:**
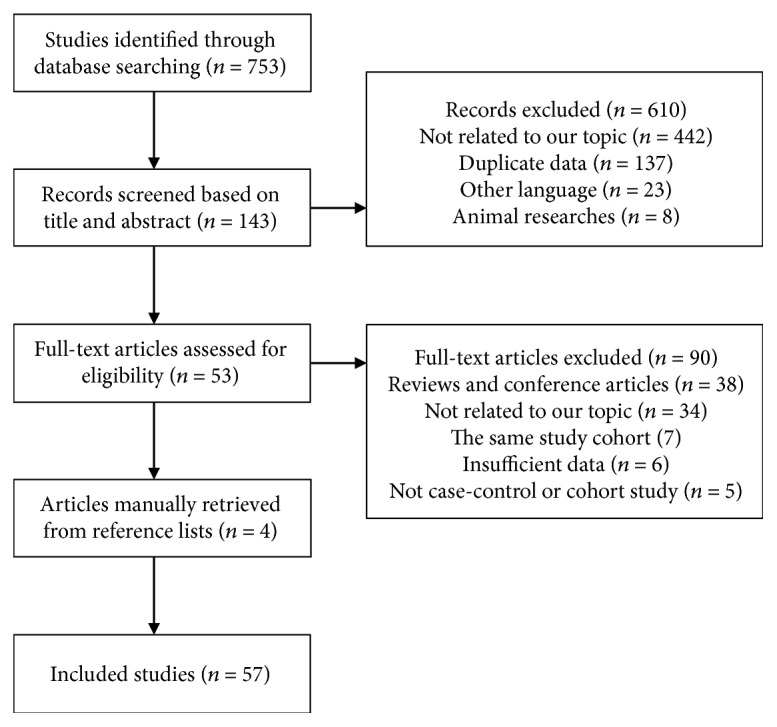
Flow chart of identification of relevant studies.

Figure 1 illustrates the results of the literature search and the process of selection. A total of 753 articles were initially identified from PubMed, Embase, and Web of Science databases. After screening based on the title and abstract, 143 articles were selected for full-text assessment; 53 studies were eligible for the final analysis. We also retrieved four relevant articles from the reference lists. Finally, 57 studies [10–66] were included in the analysis of the association of night shift work with risk of cancer.

### 3.2. Study Characteristics

The characteristics of the abovementioned studies are summarized in Table 1. Fifty-seven articles were included in this meta-analysis, including 21 case-control studies, 6 nested case-control studies, and 30 cohort studies. One article [10] included two cohorts, the Nurses' Health Study (NHS) and Nurses' Health Study II (NHS II). Therefore, a total of 58 studies were finally enrolled, involving 225,976 cases and 5,143,838 participants. We extracted information about sex in each article, except these articles that did not include classification by sex [12, 15, 48], to analyze the effect of night shift work on cancers in men and women separately. Several studies [20, 28, 43, 44, 53] analyzed the association between night shift work and different kinds of cancers. We also classified all kinds of cancer analyzed in the included studies into seven categories, including digestive system, hematological system, prostate, breast, reproductive system, lung, and skin cancers. A total 27 articles were from Europe, 11 from Asia, 17 from North America, and 3 from Australia. Most studies were based on a population with no specific occupation whereas other studies involved participants with a specific occupation, such as nurses, textile workers, women in the military, and pilots. According to the definition of night shift work, work schedules were classified as rotating shift (29 studies), fixed shift (9 studies), or mixed shift (27 studies). In fact, a cross section of studies described different work schedules, which we extracted simultaneously. Exposure assessment was performed using a questionnaire, interview, or databases. A total 43 studies were adjusted for more than four confounders and 15 studies for fewer than four confounders. The average NOS score was 7.2, and scores ranged from 4 to 8.

### 3.3. Association between Night Shift Work and the Risk of Various Cancers

**Figure 2:**
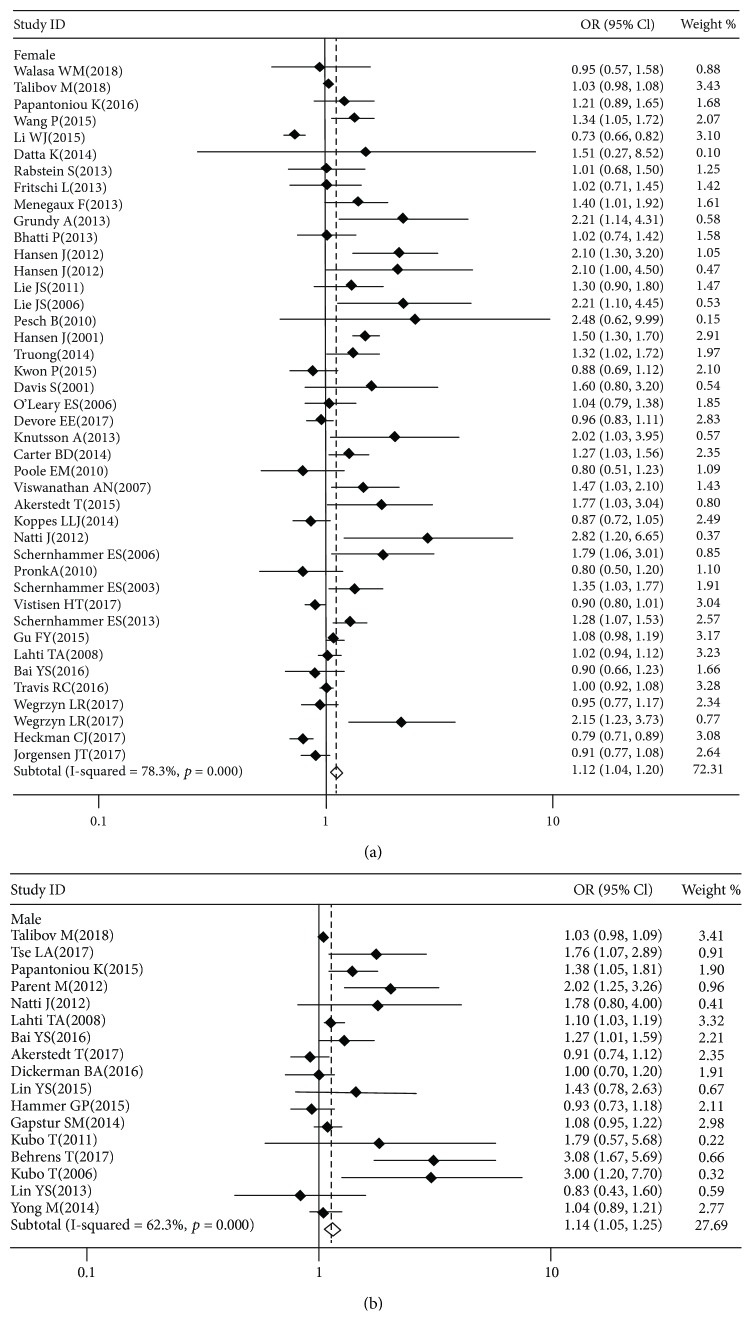
Forest plots of studies describing the association between night shift work and the risk of multiple cancers in women (a) and men (b) separately. *I* ^2^: the indicator for judging the degree of heterogeneity; OR: odds ratio; CI: confidence interval. The squares and horizontal lines represent the study-specific OR and 95% CI. The diamond represents the pooled OR and 95% CI.

The random effects model was used to pool the ORs, indicating the relationship between night shift work and risk of multiple cancers. The pooled OR was 1.15 (95% CI = 1.08–1.22, *P* < 0.001), with high heterogeneity (*I* ^2^ = 76.2%, *P* ≤ 0.001) (shown in online Figure S1). We observed that night shift work could increase the risk of cancers both in men (OR = 1.14, 95% CI = 1.05–1.25, *P* = 0.003) and women (OR = 1.12, 95% CI = 1.04–1.20, *P* = 0.002), with high heterogeneity in men (*I* ^2^ = 78.3%, *P* ≤ 0.001) and women (*I* ^2^ = 62.3%, *P* ≤ 0.001) (Figure 2). In cancers that can occur in both men and women (i.e., excluding breast, prostate, and reproductive system cancers, such as ovarian, endometrial, and testis cancer), night shift work demonstrated a positive association with the risk of cancer in men (OR = 1.09, 95% CI = 1.01–1.17, *P* = 0.031) but not in women (OR = 1.02, 95% CI = 0.94–1.12, *P* = 0.637).

### 3.4. Subgroup Analysis and Metaregression Analysis

To explore the source of potential heterogeneity and assess the influence of specific characteristics of night shift work and cancer risk, we conducted subgroup analyses, including for shift schedule, type of cancer, study region, participant occupation, study design, exposure assessment, number of adjusted variables, and NOS score (Table 2). Among the different work schedules, rotating shift work (OR = 1.14, 95% CI = 1.04–1.24) increased cancer risk whereas fixed shift work (OR = 1.09, 95% CI = 0.90–1.31) did not. A significant relationship was observed for breast cancer (OR = 1.22, 95% CI = 1.08–1.38), prostate cancer (OR = 1.26, 95% CI = 1.05–1.52), and digestive system cancer (OR = 1.15, 95% CI = 1.01–1.32). With respect to region, studies in Europe (OR = 1.18, 95% CI = 1.10–1.28) and North America (OR = 1.16, 95% CI = 1.04–1.31) showed higher ORs than those in Asia and Australia. When stratified by study design, a positive association was revealed for case-control studies (OR = 1.28, 95% CI = 1.15–1.42) and cohort studies (OR = 1.07, 95% CI = 1.00-1.15) but not nested case-control studies. For different occupations, studies based on populations in which no specific occupation was classified showed higher risk estimates (OR = 1.17, 95% CI = 1.10–1.25). Nurses (OR = 1.17, 95% CI = 1.02–1.35) had elevated cancer risk, but participants with industrial occupations did not. The interview group, which had more comprehensive information collection, presented a higher risk estimate (OR = 1.32, 95% CI = 1.17–1.49) than studies using questionnaires and databases to collect information. Regarding NOS score, studies with high-quality scores were associated with increased risk (OR = 1.14, 95% CI = 1.08–1.21) and decreased heterogeneity (*I* ^2^ = 61.6%, *P* ≤ 0.001) whereas those with low-quality scores did not show this positive relationship and had high heterogeneity (*I* ^2^ = 86.9%, *P* ≤ 0.001). Additionally, increased risk was present in studies with more than four adjusted variables. Studies with fewer than four adjusted variables showed no elevated risk of cancer, with high heterogeneity (*I* ^2^ = 82.7%, *P* ≤ 0.001). We performed metaregression analyses to assess the potential heterogeneity sources (Table 2); however, the results showed that none of the subgroups generated the potential heterogeneity.

### 3.5. Sensitivity Analysis

Sensitivity analysis showed that the pooled ORs were stable and did not identify the origins of heterogeneity. After omitting 19 studies by the leave-one-out analyses, we found a stable positive relationship (OR = 1.06, 95% CI = 1.02–1.11) between night shift work and the risk of cancer, with low heterogeneity (*I* ^2^ = 29.8%). It was found that none of the individual studies could powerfully change the positive result.

### 3.6. Dose–Response Analysis of Night Shift Work and the Risk of Cancers

**Figure 3:**
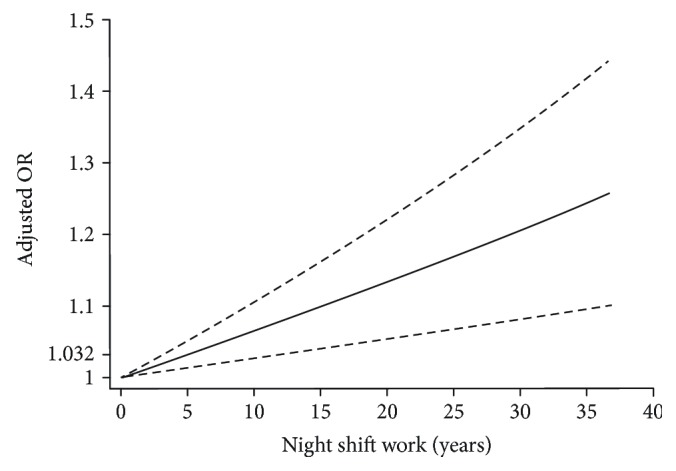
Dose–response relation plots between night shift work and the risk of multiple cancers. OR: odds ratio. Solid line represents the estimated OR by years, and the dotted lines represent the low and upper limits of 95% CIs separately.

Twenty-nine studies, which involved at least three levels of night shift exposure, were included in the dose–response analysis of night shift work and cancer risk. We used the two-stage random effects model to evaluate the linearity relationship (*P* < 0.001). For every 5 years of night shift work, the risk of cancer increased by 3.2% (OR = 1.032, 95% CI = 1.013–1.051) (Figure 3).

### 3.7. Publication Bias

**Figure 4:**
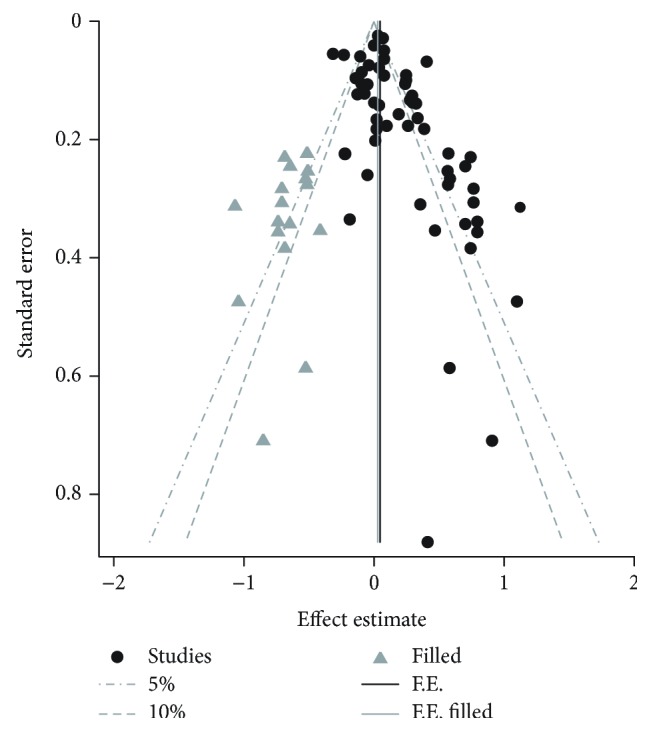
The contour-enhanced funnel plot of studies assessing the association between night shift work and the risk of cancers after using the trim and fill method.

The Begg test showed a potential publication bias among all enrolled studies (*P* = 0.001). After combining the trim and fill method and contour-enhanced funnel plot, the result showed that most of the filled studies were outside the 10% line, which indicated that the previously verified bias was mostly caused by the high heterogeneity, not the publication bias (Figure 4). The filled risk estimate was still positive, as before (OR = 1.06, 95% CI = 1.01–1.11), such that the pooled OR was stable in our study.

## 4. Discussion

This meta-analysis, consisting of 58 studies with 225,976 cases and 5,143,838 participants, revealed a positive relationship between night shift work and the risk of cancer. Compared with people who never experience working late, the risk of cancer was found to be increased by 15% in all shift workers, by 12% in female workers and 14% in male workers. A linear dose–response relationship showed a positive gradient of cancer risk with cumulative years of night shift work; for every 5 years of night shift work, cancer risk increased by 3.2%. Yuan et al. [8] confirmed that night shift work elevates the risk of multiple cancers in women, especially breast cancer. Several meta-analyses [79–81] have verified the positive relationship between night shift work and risk of prostate cancer. We obtained the same result, i.e., that long-time night shift work was associated with a higher risk of breast cancer (OR = 1.22, 95% CI = 1.08–1.38), prostate cancer (OR = 1.26, 95% CI = 1.05–1.52), and cancers in women (OR = 1.12, 95% CI = 1.04–1.20). As far as we know, this is the first meta-analysis to comprehensively explore the effect of night shift work on multiple cancers in the whole population and separately in men and women.

Tissue-specific functions and output circadian rhythms are related to the different cell-based clock genes in periphery [83]. To exclude the tissue-specific influence, we only analyzed cancers that can occur in both men and women and found that night shift work increased cancer risk in men (OR = 1.09, 95% CI = 1.02–1.17) but not in women (OR = 1.02, 95% CI = 0.94–1.12). One meta-analysis involving colorectal cancer [82] demonstrated that night shift work could increase the risk of this type of cancer in women. However, we did not find a risk relationship for either men or women based on more studies of colorectal cancer (data not shown). Although there were considerably fewer articles on other cancers than on breast and prostate cancers, the low heterogeneity for digestive system cancer (*P* = 0.081, *I* ^2^ = 40.2%), hematological system cancer (*P* = 0.066, *I* ^2^ = 54.7%), and lung cancer (*P* = 0.078, *I* ^2^ = 49.5%) presented a more reliable conclusion. Previous studies have suggested that a common mechanism might be shared among hormone-dependent cancers including prostate cancer in men and breast and ovarian cancers in women [91, 92]. Melatonin has been implicated in antiproliferation effects in vivo and in vitro, and an elevated PSA level has been strongly connected with night shift work [91, 93], which could illustrate why breast and prostate cancers are more sensitive to night shift work than other common cancers.

One meta-analysis [8] analyzing the influence of night shift work on the risk of multiple cancers in women included up to 61 articles. Although light at night (LAN) [94] has been considered one of the risk factors for cancer, studies describing LAN were not included in our meta-analysis if the analysis of LAN was not connected to night shift work. We also excluded cross-sectional studies or studies only describing sleep duration. Therefore, the exposure of all 58 studies in our article was night shift work, which could decrease the clinical heterogeneity, making a more reliable result possible. Whereas the definition of night shift work differs largely among studies, we further divided work schedules into fixed shift, rotating shift, and mixed schedule, to reduce heterogeneity. Consistent with Mancio et al. [79], rotating shift workers had evidence of a higher risk of cancer whereas no association was observed in fixed shift workers. One speculation was that constant and rapid changing work times among rotating shift workers may necessitate a severe circadian disruption, causing failed adaptation, whereas fixed night shift workers had sufficient time to adapt almost completely to the shift cycle [95]. Consequently, rotating shift work resulted in a more profound effect on carcinogenesis through severe circadian disruption.

Our subgroup analyses also uncovered other meaningful results. One finding demonstrated that prostate, breast, and digestive system cancers were connected with night shift work whereas night shift work did not raise the risk of cancers of the hematological system, reproductive system, lung, and skin. In addition, Yuan et al. [8] found that female night shift workers in Europe and North America have greater risk of cancer than women in Asia and Australia. Based on the whole population, our results were consistent with those findings and indicate that the association of cancer risk with night shift work is not largely different between men and women. The different associations might be attributed to the limitations of the study populations. Many studies from Asia were limited to industrial workers whereas most studies from Europe and North America were based on the general population. However, the contrasting results might essentially be owing to differences in ethnicity or sensitivity. More specific exploration based on ethnicity is indispensable in future research. Moreover, studies based on the general population showed a higher cancer risk than those among nurses and industrial workers, and the pooled ORs in population could be better generalized to the overall population. Cohort studies, meaning higher-quality study designs, also indicated the same positive association between night shift work and the risk of cancer. Accordingly, the higher pooled ORs in these subgroups could confirm this association more powerfully.

Through analyzing *Q* and *I* ^2^ values, we found a significant heterogeneity among the studies included in this article (*P* < 0.001, *I* ^2^ = 76.2%); therefore, we used a random effects model to decrease the heterogeneity. After subgroup analyses, we found that fixed shift work, digestive system cancer, reproductive system cancer, unclassified occupation, interview data collection, and high-quality studies were related to less heterogeneity, representing more reliable results. However, all *P* values in the metaregression analyses did not reflect a statistical difference, such that heterogeneity could not be explained by metaregression analysis. One-by-one-omitted sensitivity and leave-one-out analyses showed that the pooled risk estimates were stable and positive, even when 19 studies were omitted, until heterogeneity was reduced to 29.8%. Although we did not find an obvious source of heterogeneity, the specific subgroup analyses, such as a more uniform definition of work schedules, unclassified occupation based on population, more detailed interviews, and high-quality studies, could decrease the potential heterogeneity.

Theoretical biological mechanisms for the positive relationship between night shift work and cancer risk are complex. First, night shift work and LAN could disturb the normal synchrony with the day–night rhythm and sleeping and diet patterns and bring about circadian disruption, which could suppress the secretion of melatonin [80]. Melatonin plays a pivotal role in inhibiting carcinogenesis through antioxidation, regulation of the immune system, free radical scavenging, and antiangiogenesis [67]. Decreased melatonin levels might disturb its antiproliferation effects on prostate cancer cells both in vivo and in vitro [93] and induce continuous secretion of estrogen, to increase the risk of breast cancer [77]. Second, night shift work can reduce the exposure time to sunlight and subsequently decrease vitamin D levels [80]. Studies have supported the inverse association between circulating vitamin D levels and risk of breast [96], colorectal [97], and prostate cancer [98]. Third, from a molecular perspective, night shift work could constitute a disruption of the feedback loops of circadian genes and lead to subsequent disordered expression of transcription and translation in all cells, which could pose a threat to cell proliferation, metabolism, regulation of the immune system, and DNA damage repair, causing carcinogenesis [73, 74].

To the best of our knowledge, this meta-analysis is the first and most comprehensive of its kind to identify the association between night shift work and risk of cancer from the perspective of diverse cancers and by sex. There were several strengths in our meta-analysis. First, we enrolled a large number of articles, even using strict inclusion criteria. The massive study population could enhance statistical power and ensure more accurate risk estimation. Second, a linear dose–response analysis was used to quantify the association between accumulative years of night shift work and cancer risk. Third, we classified work schedules and found that rotating shift work could increase cancer risk whereas fixed shift work could not. The classification of work schedules could decrease clinical heterogeneity to make the results more reliable. Fourth, 34 of 57 studies were carried out among the general population, such that the pooled OR could be better extended to the entire population. Our meta-analysis also had several limitations. First, a significantly high heterogeneity was discovered. We observed significant variability in the study design, risk estimates, study population, definition of night shift work, and exposure assessment. Each of these aspects may generate heterogeneity. Even though many statistical methods were used, we still had trouble finding an obvious source of potential heterogeneity; therefore, the conclusions reached in our meta-analysis should be interpreted with caution. Second, the lack of a consistent definition of night shift work may lead to a certain degree of misclassification and result in a dilution of the pooled OR [8]. Third, given that most night shift workers tend to have lower socioeconomic status, a lower uptake of screening and response rates may result in underestimation of the pooled risk estimates. Finally, studies using interviews could actively collect more detailed information, presenting a stronger risk compared with studies using questionnaire- and database-based data collection. In addition, there is inherent recall bias when conducting interviews or questionnaires. Hence, different exposure assessment methods and studies with lower quality or a less number of adjusted variables can cause information bias.

In conclusion, our meta-analysis identified a positive relationship between night shift work and cancer risk, using a comprehensive perspective of common cancers. We revealed that the risk of cancer increases cumulatively by 3.2% for every 5 years of night shift work. Moreover, we found no difference between men and women in the association between night shift work and the risk of cancer. Overall, on the grounds that public health is adversely affected by night shift work and its prevalence is on the rise, it is indispensable to develop shift work schedules with the aim of reducing cancer risk. Our meta-analysis does not merely increase public awareness, it also supports the recommendation for regular cancer screening among night shift workers.

## Figures and Tables

**Table 1:** Characteristics of studies included in meta-analysis.

Study (year)	Region	Study design	No. of cases	Occupation	Exposure	Adjusted OR (95% CI)	Type of cancer	Adjusted item	Exposure assessment method
Walasa et al. (2018) [40]	Australia	Case-control study	350	NA	Night, never vs. 7.5+ years	0.95 (0.57–1.58)	Colorectal cancer	Age group, education level, socioeconomic status, lifetime cigarette smoking, and alcohol intake 10 years ago	Questionnaire
Talibov et al. (2018) [41]	Europe	Case-control study	131,594	NA	Rotating, never vs. 20+ years	1.033 (0.984–1.084)	Hematological system cancer	Cumulative benzene, formaldehyde, and ionizing radiation	Questionnaire
Tse et al. (2017) [11]	Asia	Case-control study	431	NA	Night, never vs. ever	1.76 (1.07–2.89)	Prostate cancer	Age at interview, marital status, unemployment status, family prostate cancer history, consumption of deep fried food, consumption of pickled vegetable, green tea drinking habits, and cbpai	Interview
Heckman et al. (2017) [44]	North America	Cohort study	4854	Nurses	Rotating, never vs. 10+ years	0.794 (0.711–0.888)	Skin cancer	Years of shift work, hours of sleep, sleep adequacy, sleepy days per week, snoring, restless legs syndrome, family history of melanoma, hours spent in sun, number of severe sunburns, sunburn severity, artificial tanning frequency, annual uv at residence, moles on lower legs, natural hair color in adolescence, marital status, financial status, BMI, physical activity, smoking status, menopausal status, postmenopausal hormones, oral contraceptive use, and healthy eating index	Questionnaire
Papantoniou (2017) [12]	Europe	Case-control study	1626	NA	Night and rotating, never vs. 15+ years	1.28 (1.06–1.56)	Colorectal cancer	Age, centre, educational level, sex, history of colorectal cancer, BMI, smoking, leisure time physical activity, alcohol consumption, total energy intake in grams/day, all red meat consumption, sleep duration, bisphosphonates, and NSAIDs	Questionnaire
Devore et al. (2017) [45]	North America	Cohort study	3014	Nurses	Rotating, never vs. 10+ years	0.96 (0.83, 1.11)	Colorectal adenoma	Age, time period of first lower endoscopy, reason for endoscopy, family history of cancer, height, BMI, physical activity, smoking, alcohol intake, menopausal status, menopausal hormone use, oral contraceptive use, multivitamin use, total calcium intake, vitamin d intake, red meat intake, NSAIDs use, and predicted vitamin D score	Questionnaire
Vistisen et al. (2017) [42]	Europe	Cohort study	1245	NA	Night, never vs. ever	0.90 (0.80–1.01)	Breast cancer	Calendar year, age, age at birth of first child, number of births, family history of breast cancer or ovarian cancer, oral contraception, hormone replacement therapy, other sex hormones, medication related to alcoholism, mammography screening attendance, and highest family educational level	Database
Wegrzyn et al. (2017)^a^ [10]	North America	Cohort study	5971	Nurses	Rotating, never vs. 30+ years	0.95 (0.77–1.17)	Breast cancer	Age, height, BMI, BMI at age 18, adolescent body size, age at menarche, age at first birth and parity combined, breast feeding, type of menopause and age at menopause, combined menopausal hormone therapy, duration of estrogen alone menopausal hormone therapy, duration of estrogen and progesterone menopausal hormone therapy, first-degree family history of breast cancer, history of benign breast disease, alcohol consumption, physical activity, and current mammography use.	Questionnaire
Wegrzyn et al. (2017)^a^ [10]	North America	Cohort study	3570	Nurses	Rotating, never vs. 20+ years	2.15 (1.23–3.73)	Breast cancer	Age, height, BMI, BMI at age 18, adolescent body size, age at menarche, age at first birth and parity combined, breast feeding, type of menopause and age at menopause, combined menopausal hormone therapy, duration of estrogen alone menopausal hormone therapy, duration of estrogen and progesterone menopausal hormone therapy, first-degree family history of breast cancer, history of benign breast disease, alcohol consumption, physical activity, and mammography use.	Questionnaire
Jørgensen et al. (2017) [43]	Europe	Cohort study	945	Nurses	Rotating, night, and rotating, never vs. ever	0.91 (0.77–1.08)	Unclassified cancer	Age, smoking, pack-years, physical activity, BMI, alcohol consumption, diet (vegetables, fruit, and fatty meat consumption), preexisting diseases (hypertension, diabetes, and myocardial infarction), self-reported health, stressful work environment, marital status, female reproductive factors (birth, use of hormone therapy, and oral contraceptives)	Questionnaire
Behrens et al. (2017) [13]	Europe	Cohort study	76	NA	Rotating, never vs. 20+ years	3.08 (1.67–5.69)	Prostate cancer	Age, smoking, family history of prostate cancer, level of school education, and equivalent income	Interview
Akerstedt et al. (2017) [46]	Europe	Cohort study	454	NA	Night, never vs. ever	0.91 (0.74–1.12)	Prostate cancer	Age, education level, tobacco consumption, BMI, having children, coffee consumption, and previous cancer	Interview
Dickerman et al. (2016) [49]	Europe	Cohort study	602	NA	Rotating, never vs. ever	1.0 (0.7–1.2)	Prostate cancer	Age, education, BMI, physical activity, social class, smoking status, alcohol use, snoring, and zygosity	Questionnaire
Papantoniou et al. (2016) [47]	Europe	Case-control study	1708	NA	Night and rotating, never vs. 15+ years	1.21 (0.89–1.65)	Breast cancer	Age, centre, educational level, parity, menopausal status, family history of breast cancer, BMI, smoking status, oral contraceptive use, leisure time physical activity, alcohol consumption, and sleep duration	Interview
Gyarmati et al. (2016) [48]	Europe	Case-control study	374	NA	Night and rotating, never vs. 20+ years	1.1 (0.8–1.6)	Stomach cancer	Age, sex, educational level, centre, BMI, cigarette smoking status, family history, physical activity level, total energy intake, grams of red meat, grams of vegetables, and grams of fruit and alcohol consumption	Interview
Costas et al. (2016) [15]	Europe	Case-control study	321	NA	Night, never vs. 20+ years	1.77 (1.14–2.74)	Chronic lymphocytic leukemia	Region, age, sex, worked on a farm, family history of hematologic malignancies, BMI, tobacco consumption (never, past, and current), sleep problems, and education	Interview
Bai et al. (2016) [16]	Asia	Cohort study	1251	NA	Night, never vs. 20+ years	1.08 (0.90–1.29)	Unclassified cancer	Age, BMI, family history of cancer, alcohol drinking and smoking status, number of children, menopausal status, hormone replacement therapy, and contraception status	Questionnaire
Travis et al. (2016) [14]	Europe	Cohort study	4809	NA	Night, never vs. ever	1.00 (0.92–1.08)	Breast cancer	Socioeconomic status, parity and age at first birth, BMI, alcohol intake, strenuous physical activity, family history of breast cancer, age at menarche, oral contraceptive use, smoking, living with a partner, and hormone therapy	Questionnaire
Gu et al. (2015) [20]	North America	Cohort study	5413	Nurses	Rotating, never vs. 15+ years	1.08 (0.98–1.19)	Unclassified cancer	Age, alcohol consumption, physical exercise, multivitamin use, menopausal status and postmenopausal hormone use, physical exam in the past 2 years, healthy eating score, smoking status, pack-years, BMI, and husband's education	Questionnaire
Wang P. et al. (2015) [17]	Asia	Case-control study	712	NA	Night, never vs. ever	1.34 (1.05–1.72)	Breast cancer	Age, education, BMI, age at menarche, menopausal status, parity, physical activity, breast feeding, and family history of cancer	Interview
Papantoniou et al. (2015) [18]	Europe	Case-control study	1115	NA	Night and rotating, never vs. 28+ years	1.38 (1.05–1.81)	Prostate cancer	Age, centre, educational level, family history of prostate cancer, physical activity over the past decade, smoking status, past sun exposure, and daily meat consumption	Interview
Li et al. (2015) [51]	Asia	Nested case-control study	1709	Industry	Night, never vs. ever	0.73 (0.66–0.82)	Breast cancer	Age at the beginning of follow-up	Questionnaire
Lin et al. (2015) [50]	Asia	Cohort study	94	NA	Rotating, never vs. ever	1.43 (0.78–2.63)	Biliary tract cancer	Age, BMI, history of cholelithiasis, history of diabetes, cigarette smoking, alcohol drinking, perceived stress, and sleep time	Questionnaire
Kwon et al. (2015) [52]	Asia	Nested case-control study	1451	Industry	Rotating, never vs. 30.6+ years	0.88 (0.69–1.12)	Lung cancer	Adjusted for age, smoking, parity, and endotoxin	Database
Akerstedt et al. (2015) [21]	Europe	Cohort study	463	NA	Night, never vs. 21+ years	1.77 (1.03–3.04)	Breast cancer	Age, education level, tobacco consumption, BMI, having children, coffee consumption, previous cancer, and use of hormones including oral contraceptives	Interview
Hammer et al. (2015) [19]	Europe	Cohort study	337	Industry	Rotating, never vs. ever	0.93 (0.73–1.18)	Prostate cancer	Age and professional status	Database
Gapstur et al. (2014) [55]	North America	Cohort study	4974	NA	Rotating, night, and evening, never vs. ever	1.08 (0.95–1.22)	Prostate cancer	Age, race, education, BMI, smoking status, family history of prostate cancer, and painful/frequent urination	Questionnaire
Koppes et al. (2014) [54]	Australia	Cohort study	2531	NA	Night, never vs. ever	0.87 (0.72–1.05)	Breast cancer	Night work, age, origin, children in household, education, occupation, job tenure (years), and contractual working hours	Interview
Carter et al. (2014) [23]	North America	Cohort study	1289	NA	Rotating, night, and evening, never vs. ever	1.27 (1.03–1.56)	Ovarian cancer	Oral contraceptive use, age at menarche and menopause, tubal ligation, parity, postmenopausal estrogen use, race, family history of cancers, exercise, BMI, and height	Questionnaire
Yong et al. (2014) [53]	Europe	Cohort study	10,873	Industry	Rotating, never vs. ever	1.04 (0.89–1.21)	Unclassified cancer	Age, job level, cigarette smoking, and employment duration in categories	Questionnaire
Datta et al. (2014) [56]	Asia	Case-control study	50	Industry	Night, never vs. ever	1.51 (0.27–8.52)	Breast cancer	None	Interview
Truong et al. (2014) [22]	Europe	Case-control study	1126	NA	Night, never vs. ever	1.32 (1.02–1.72)	Breast cancer	Age, study area, parity, age at first full-term pregnancy, age at menarche, family history of breast cancer, current use of hormonal replacement therapy, BMI, tobacco, and alcohol	Interview
Knutsson et al. (2013) [26]	Europe	Cohort study	94	NA	Night, never vs. ever	2.02 (1.03–3.95)	Breast cancer	Height, weight, waist, hip circumference, educational level, number of children, smoking, menopausal status, oral contraceptive use, hormones other than contraceptives, alcohol intake, educational level, BMI, and waist–hip ratio	Questionnaire
Rabstein et al. (2013) [57]	Europe	Case-control study	857	NA	Night, never vs. ever	1.01 (0.68–1.5)	Breast cancer	Age, adjusted for family history of breast cancer, hormone replacement use, and number of mammograms	Interview
Fritschi et al. (2013) [59]	Australia	Case-control study	1205	NA	Night, never vs. 20+ years	1.02 (0.71–1.45)	Breast cancer	Light at night, phase shift and sleep disruption, poor diet, lack of physical activity and little time outdoors, and age	Questionnaire
Schernhammer et al. (2013) [24]	North America	Cohort study	1455	Nurses	Rotating, never vs. 15+ years	1.28 (1.07–1.53)	Lung cancer	Age, smoking status, age at start of smoking, cigarettes smoked per day among current smoker, time since quitting among past smokers, fruit intake, vegetable intake, BMI, and environmental smoking exposures	Questionnaire
Menegaux et al. (2013) [25]	Europe	Case-control study	1232	NA	Night, never vs. 4.5+ years	1.40 (1.01–1.92)	Breast cancer	Age, study area, parity, age at first full-term pregnancy, age at menarche, family history of breast cancer, current hormonal replacement therapy, BMI, tobacco, and alcohol	Interview
Grundy et al. (2013) [27]	North America	Case-control study	1134	NA	Mixed, never vs. 30+ years	2.21 (1.14–4.31)	Breast cancer	Age and centre	Questionnaire
Bhatti et al. (2013) [60]	North America	Case-control study	1490	NA	Night, never vs. 7+ years	1.02 (0.74–1.42)	Ovarian cancer	Age at reference, county, reference year, duration of oral contraceptive use, number of full-term pregnancies, and BMI	Interview
Lin et al. (2013) [58]	Asia	Cohort study	127	Industry	Rotating, never vs. ever	0.83 (0.43–1.60)	Pancreatic cancer	Age, BMI, history of diabetes, alcohol drinking, cigarette smoking, perceived stress, and sleep time	Questionnaire
Hansen and Stevens (2012) [30]	Europe	Nested case-control study	267	Nurses	Night and evening, never vs. 20+ years	2.1 (1.3–3.2)	Breast cancer	Adjusted for age, weight regularity, use of hormone replacement therapy, age at menarche, menstrual regularity, menopausal status, age at birth of first child, breast cancer in mother or sister, and total duration of lactation	Interview
Hansen and Lassen (2012) [31]	Europe	Nested case-control study	132	Industry	Evening, never vs. 15+ years	2.1 (1.0–4.5)	Breast cancer	Adjusted for age, hormone replacement therapy, number of childbirths, age at menarche, years of education, occasional sunbathing frequency, and tobacco smoking status	Questionnaire
Parent et al. (2012) [28]	North America	Case-control study	3137	NA	Night, never vs. 10+ years	2.016 (1.246–3.261)	Unclassified cancer	None	Interview
Natti et al. (2012) [29]	Europe	Cohort study	99	NA	Night, never vs. ever	2.148 (1.178–3.917)	Unclassified cancer	Age, longstanding illness (among men), and smoking status	Interview
Kubo et al. (2011) [32]	Asia	Cohort study	17	Industry	Rotating, never vs. ever	1.79 (0.57–5.68)	Prostate cancer	Age, BMI, alcohol intake, smoking, exercise, and marital status	Database
Lie et al. (2011) [62]	Europe	Nested case-control study	699	Nurses	Night, never vs. 12+ years	1.3 (0.9–1.8)	Breast cancer	Age, period of diagnosis, parity, family history of breast cancer in mother or sister, and frequency of alcohol consumption at time of diagnosis	Interview
Poole et al. (2011) [61]	North America	Cohort study	718	Nurses	Rotating, never vs. 20+ years	0.8 (0.51–1.23)	Ovarian cancer	Age, duration of oral contraceptive use, parity, BMI, smoking status, tubal ligation history, menopausal status, family history of ovarian cancer (yes/no), and duration of breastfeeding	Questionnaire
Pesch et al. (2010) [64]	Europe	Case-control study	753	NA	Night, never vs. 20+ years	2.48 (0.62–9.99)	Breast cancer	Age in 5-year groups, adjusted for family history of breast cancer, hormone replacement use, and number of mammograms	Interview
Pronk et al. (2010) [63]	Asia	Cohort study	349	NA	Mixed, never vs. 17+ years	0.8 (0.5–1.2)	Breast cancer	Age, education, family history of breast cancer, number of pregnancies, age at first birth, and physical activity	Interview
Lahti et al. (2008) [33]	Europe	Cohort study	6307	NA	Rotating, never vs. ever	1.07 (1.01–1.13)	Non-Hodgkin lymphoma	Age, social class, and cohort period	Database
Viswanathan et al. (2007) [34]	North America	Cohort study	515	Nurses	Rotating, never vs. 20+ years	1.47 (1.03–2.10)	Endometrial cancer	Age, age at menarche, age at menopause, parity, BMI, oral contraceptive use, use and duration of postmenopausal hormones, hypertension, diabetes, and smoking	Questionnaire
Lie et al. (2006) [36]	Europe	Nested case-control study	537	Nurses	Rotating, never vs. 30+ years	2.21 (1.10–4.45)	Breast cancer	Total employment time as a nurse and parity	Database
O'Leary et al. (2006) [65]	North America	Case-control study	487	NA	Mixed, never vs. ever	1.04 (0.79–1.38)	Breast cancer	Age at reference date, parity, family history, education, and history of benign breast disease	Interview
Schernhammer et al. (2006) [35]	North America	Cohort study	1352	Nurses	Rotating, never vs. 20+ years	1.79 (1.06–3.01)	Breast cancer	Age, age at menarche, menopausal status, age at menopause, age at first birth, BMI, current alcohol consumption, oral contraceptive use, postmenopausal hormone use, smoking status, benign breast disease, family history of breast cancer, and physical activity	Questionnaire
Kubo et al. (2006) [37]	Asia	Cohort study	31	Industry	Rotating, never vs. ever	3.0 (1.2–7.7)	Prostate cancer	Age, study area, family history of prostate cancer, BMI, smoking, alcohol drinking, job type, physical activity at work, workplace, perceived stress, educational level, and marriage status	Questionnaire
Schernhammer et al. (2003) [38]	North America	Cohort study	602	Nurses	Rotating, never vs. 15+ years	1.35 (1.03–1.77)	Colorectal cancer	Age in years, smoking, BMI, physical activity in quintiles, regular aspirin use, colorectal cancer in parent or sibling, screening endoscopy during the study period, consumption of beef, pork, or lamb as a main dish, alcohol consumption status, total caloric intake in quintiles, use of postmenopausal hormones, menopausal status, and height in seven categories	Questionnaire
Davis et al. (2001) [66]	North America	Case-control study	767	NA	Mixed, never vs. 3+ years	1.6 (0.8–3.2)	Breast cancer	Parity, family history of breast cancer (mother or sister), oral contraceptive use (ever), and recent (<5 years) discontinued use of hormone replacement therapy	Interview
Hansen (2001) [39]	Europe	Case-control study	6281	NA	Night, never vs. 0.6+ years	1.5 (1.3–1.7)	Breast cancer	Age, social class, age at birth of first child, age at birth of last child, and number of children	Interview

Abbreviations: BMI: body mass index; NSAIDs: nonsteroidal anti-inflammatory drugs; NA: not available. ^a^This study included two prospective cohorts (NHS and NHS2).

**Table 2:** The results of subgroup analyses and metaregression analyses on the association between night shift work and the risk of cancers.

Subgroup	No. of studies	Weight (%)	OR (95% CI)	*I* ^2^	*P* for heterogeneity	*P* ^∗^ for interaction
Shift schedule^a^						0.570
Rotating shift	29	46.97	1.14 (1.04–1.24)	68.7%	<0.001	
Fixed shift	9	11.19	1.09 (0.90–1.31)	51.1%	0.037	
Mixed shift	27	41.84	1.20 (0.82–1.77)	80.7%	<0.001	
Type of cancer^b^						0.298
Digestive system cancer	11	15.72	1.15 (1.01–1.32)	40.2%	0.081	
Hematological system cancer	5	9.12	1.08 (0.99–1.17)	54.7%	0.066	
Prostate cancer	11	16.10	1.26 (1.05–1.52)	73.2%	<0.001	
Breast cancer	37	39.62	1.22 (1.08–1.38)	81.2%	<0.001	
Reproductive system cancer	6	7.99	1.06 (0.85–1.32)	49.5%	0.078	
Lung cancer	5	7.53	1.08 (0.87–1.35)	53.4%	0.073	
Skin cancer	3	3.92	0.93 (0.50–1.74)	74.9%	0.019	
Region						0.298
Australia	3	5.03	0.91 (0.77–1.06)	0.0%	0.728	
Europe	27	48.92	1.18 (1.10–1.28)	75.1%	<0.001	
Asia	11	14.29	1.11 (0.88–1.39)	78.3%	<0.001	
North America	17	31.75	1.16 (1.04–1.31)	76.1%	<0.001	
Occupation						0.795
Unclassified occupation	35	61.45	1.17 (1.10–1.25)	69.7%	<0.001	
Industry	9	12.09	1.00 (0.81–1.24)	72.8%	<0.001	
Nurses	14	26.46	1.17 (1.02–1.35)	80.6%	<0.001	
Study design						0.845
Case-control study	21	32.23	1.28 (1.15–1.42)	66.5%	<0.001	
Nested case-control study	6	9.08	1.30 (0.89–1.90)	88.0%	<0.001	
Cohort study	31	58.69	1.07 (1.00–1.15)	70.9%	<0.001	
Exposure assessment						0.075
Questionnaire	28	53.99	1.08 (1.00–1.17)	77.1%	<0.001	
Interview	24	34.52	1.32 (1.17–1.49)	66.3%	<0.001	
Database	6	9.06	1.00 (0.85–1.18)	77.1%	0.004	
Number of adjusted variables						0.926
≤4	15	22.84	1.13 (0.99–1.28)	82.7%	<0.001	
>4	43	77.16	1.16 (1.08–1.24)	72.8%	<0.001	
Study score						0.585
Low quality	17	25.20	1.16 (0.98–1.37)	86.9%	<0.001	
High quality	41	74.80	1.14 (1.08–1.21)	61.6%	<0.001	

^a,b^Five studies report their studies including different kinds of cancer; nine articles report their studies including different types of shift schedules. ^∗^ *P* values for metaregression.

## Supplementary Materials

Supplementary Materials Figure S1: forest plots of studies describing the association between night shift work and the risk of multiple cancers. *I* ^2^: the indicator for judging the degree of heterogeneity; OR: odds ratio; CI: confidence interval. The squares and horizontal lines represent the study-specific OR and 95% CI. The diamond represents the pooled OR and 95% CI.
