# Supplementary figures and images for: Sex Differences in the Association between Night Shift Work and the Risk of Cancers: A Meta-Analysis of 57 Articles
Source: Dis Markers. 2018 Nov 26;2018:7925219. doi: 10.1155/2018/7925219 (PMC6287141; doi:10.1155/2018/7925219)

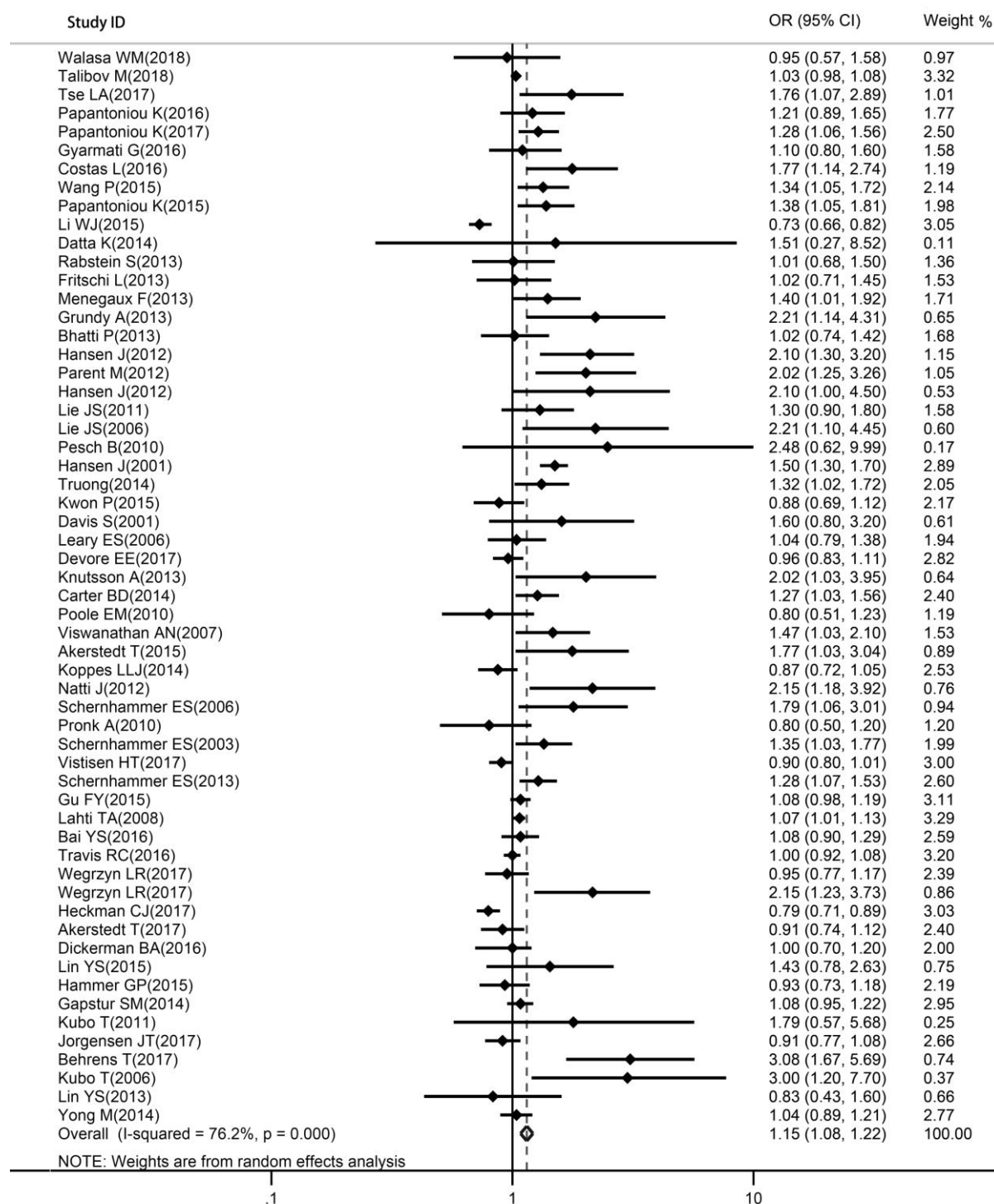

Supplementary Figure 1

Supplement: Supplementary Materials — Figure S1: forest plots of studies describing the association between night shift work and the risk of multiple cancers. I 2: the indicator for judging the degree of heterogeneity; OR: odds ratio; CI: confidence interval. The squares and horizontal lines represent the study-specific OR and 95% CI. The diamond represents the pooled OR and 95% CI. [file 7925219.f1.pdf]
